# Ionizing radiation expands a p90RSK-activated patrolling monocyte subset: modulation by colchicine

**DOI:** 10.3389/fcvm.2026.1763490

**Published:** 2026-04-15

**Authors:** Masaki Imanishi, Venkata S. K. Samanthapudi, Nhat-Tu Le, Luis Antonio Rivera, Jung Hyun Kim, Jonghae Lee, Gilbert F. Mejia, Oanh Hoang, Anita Deswal, Keri L. Schadler, Michelle A. T. Hildebrandt, Syed Wamique Yusuf, Guangyu Wang, Jared K. Burks, Roza I. Nurieva, Nicolas L. Palaskas, Kevin T. Nead, El-ad David Amir, Efstratios Koutroumpakis, Steven H. Lin, Jun-ichi Abe, Sivareddy Kotla

**Affiliations:** 1 Department of Cardiology, The University of Texas MD Anderson Cancer Center, Houston, TX, United States; 2 Department of Cardiovascular Sciences, Houston Methodist Research Institute, Houston, TX, United States; 3 Instituto Tecnológico y de Estudios Superiores de Monterrey, Escuela de Medicina y Ciencias de la Salud, Monterrey, Nuevo León, México; 4 Department of Pediatric Research, The University of Texas MD Anderson Cancer Center, Houston, TX, United States; 5 Department of Lymphoma/Myeloma, The University of Texas MD Anderson Cancer Center, Houston, TX, United States; 6 Department of Leukemia, Division of Center Medicine, The University of Texas MD Anderson Cancer Center, Houston, TX, United States; 7 Department of Leukemia, Division of Discovery Science, The University of Texas MD Anderson Cancer Center, Houston, TX, United States; 8 Department of Epidemiology, The University of Texas MD Anderson Cancer Center, Houston, TX, United States; 9 Astrolabe Diagnostics, Inc., Fort Lee, NJ, United States; 10 Department of Radiation Oncology, The University of Texas MD Anderson Cancer Center, Houston, TX, United States, United States

**Keywords:** cardiovascular disease, CHIP, colchicine, ionizing radiation (IR), p90RSK, peripheral blood mononuclear cell (PBMCs)

## Abstract

**Background:**

Ionizing radiation (IR) is known to induce vascular injury and alter immune cell function, yet the molecular mechanisms driving these changes remain incompletely defined. In particular, the role of clonal hematopoiesis-associated proteins expression and stress-responsive signaling pathways in monocyte subsets has not been fully elucidated.

**Methods:**

Peripheral blood mononuclear cells (PBMCs) from a single healthy human donor were pre-treated with colchicine and exposed to 2 Gy IR. After 24 h, cells were analyzed using CyTOF with a comprehensive antibody panel targeting lineage markers, DNA damage response proteins, efferocytosis receptors, and clonal hematopoiesis of indeterminate potential (CHIP) associated proteins, including DNMT3A and TET2. Data were processed using the Astrolabe platform to annotate canonical immune subsets.

**Results:**

While IR did not alter the overall immune cell frequency, colchicine increased the relative abundance of myeloid and B cell populations. Within CD14^−^CD16^+^ monocytes, IR enhanced p90RSK activation despite reductions in proliferation and inflammatory signaling markers (e.g., Ki67, p-JAK, p-PKC*ζ*). At the molecular level, IR reduced expression of CHIP associated proteins DNMT3A and TET2, coinciding with elevated p90RSK activity specifically in the CD14^−^CD16^+^CD68^hi^ subset. This unique patrolling monocyte population exhibited the highest p90RSK activation and expanded significantly following IR exposure-a response not observed in other monocyte subsets. Colchicine effectively suppressed this IR-induced expansion without restoring DNMT3A or TET2 expression, indicating selective modulation of p90RSK-driven signaling.

**Conclusion:**

Colchicine counteracts IR-induced immune remodeling by selectively inhibiting the expansion of CD14^−^CD16^+^CD68^hi^ monocytes, a subset defined by reduced CHIP asscoaited proteins expression and heightened p90RSK activation. These findings suggest a novel mechanism by which colchicine may modulate radiation-responsive inflammatory signaling in monocytes *in vitro*, supporting a testable hypothesis that colchicine could mitigate vascular inflammation and ultimately reduce cardiovascular risk in cancer survivors.

## Introduction

Radiation-induced cardiovascular disease (RICVD) is increasingly recognized as a major long-term complication among cancer survivors (1), particularly those treated for thoracic malignancies such as breast cancer (2–4), lung cancer (5, 6), and lymphoma (7–10). Although radiation therapy (RT) is essential for controlling these cancers, its delayed effects on the cardiovascular system contribute significantly to morbidity and mortality, often manifesting more than five years after treatment (11). For example, patients with Hodgkin lymphoma who receive chest irradiation face a sevenfold increased risk of developing cardiovascular disease (CVD), and breast cancer survivors experience a linear increase in major coronary events-approximately 7.4% per Gray (Gy) of cardiac radiation exposure. Despite improvements in RT techniques, incidental cardiac doses of 1–5 Gy remain common (1). CVD risk in cancer survivors is further compounded by elevated levels of body mass index, C-reactive protein, and LDL cholesterol, along with a phenotype of accelerated cellular senescence (12–14). Genotoxic therapies, including radiation and chemotherapy, induce mitochondrial dysfunction and persistent DNA damage, particularly at telomeres, which can last for months and drive the senescence-associated secretory phenotype (SASP). While most DNA damage is repaired within 24 h, telomeric damage persists and may underlie the delayed onset of CVD in this population (15–17). These insights highlight the need for deeper mechanistic understanding and the development of targeted strategies to prevent and treat RICVD, ultimately improving long-term outcomes for cancer survivors.

The cardiovascular benefits of colchicine have been well established through two landmark clinical trials-LoDoCo2 (18) and COLCOT (19)-which demonstrated that adding low-dose colchicine (0.5 mg daily) to standard therapy significantly reduces major adverse cardiovascular events (MACE) in patients with either chronic coronary artery disease or recent myocardial infarction. While colchicine is known to exert anti-inflammatory effects primarily through inhibition of the NLRP3 inflammasome (20), its impact on radiation-induced senescence and the SASP, key contributors to RICVD, remains underexplored. In this study, we investigated colchicine's effects on immune cell remodeling following IR *in vitro*. Notably, colchicine suppressed the IR-induced expansion of a distinct patrolling monocyte subset (CD14^−^CD16^+^CD68^hi^), which exhibited markedly elevated p90RSK activation and reduced expression of CHIP-associated proteins DNMT3A and TET2. This response was unique to this subset and was not observed in other monocyte populations. These findings suggest that colchicine modulates radiation-responsive signaling beyond its canonical anti-inflammatory role by selectively targeting p90RSK-driven pathways in CD14^−^CD16^+^CD68^hi^ monocytes. In summary, these findings establish a mechanistic framework and support further investigation of effects in models of vascular inflammation or in patients undergoing radiotherapy, with particular emphasis on elucidating mechanisms that may contribute to cardiovascular risk in cancer survivors.

## Materials and methods

### Panel design and metal conjugation of antibodies

We generated a CyTOF panel which includes antibodies against CHIP drivers associated proteins, SASP-related proteins, and various cell surface markers for PBMCs such as the markers described in the previous report (21). We used 29 antibodies, Ir DNA-Intercalator (Cell-ID Intercalator-Ir; Fluidigm #201192A) for nucleus staining and Rh DNA-intercalator (Cell-ID Intercalator-103Rh; Fluidigm #201103A) for viability staining, as listed in Table 1. The antibodies listed in Table 1, except for the metal-conjugated antibodies that were purchased from Fluidigm, BioLegend, R&D and CST, were conjugated to lanthanides using the Maxpar X8 Multimetal Labeling Kit (Fluidigm) according to the manufacturer's protocol. Although our CyTOF panel contained poly ADP-ribose polymerase (PAR) (abcam #ab14460) channel, we ignored any results derived from its signal and removed it from the heatmaps because the PAR antibody can react with BSA during the antibody conjugation step.

**Table 1:** CyTOF antibody panel and the markers with each protein function. To establish our CyTOF antibody panel, some cell surface markers were picked from the previous report (21).

Metal	Marker	Vendor	Catalog#	Surface/intracellular
139La	CD45	BioLegend	304002	S
141Pr	phospho-P90RSK(S380)	ABclonal	AP0562	I
142Nd	CD19	DVS-Fluidigm	3142001B	S
143Nd	CD11b	DVS-Fluidigm	3143015B	S
147Sm	TOP2b	R&D	MAB6348 (100 ug)	I
148Nd	Trx	Proteintech	14999-1-AP (150 uL)	I
150Nd	CD61	DVS-Fluidigm	3150001B	S
151Eu	phospho-TERF2IP(S205)	(Home made)		I
152Sm	JAK2	Abcam	ab170718 (100 ug)	I
153Eu	Cleaved caspase 3	CST	9579BF	I
154Sm	TERF2IP	Abcam	ab14404 (100 ug)	I
155Gd	IL-1b	biorbyt	orb308551 (200ug)	I
156Gd	ERK5	Abcam	ab232538 (100 ug)	I
159Tb	P90RSK	StressMarq	SPC-147F	I
160Gd	CD14	DVS-Fluidigm	3160001B	S
161Dy	phospho-ERK5 S496	Abnova	PAB15918 (100 ug)	I
162Dy	p16	Abcam	ab54210 (100 uL)	I
163Dy	CD45RA	BioLegend	304102	S
164Dy	Tyro3	Novus	NBP1-28635 (0.1 mL)	I
165Ho	P53	R&D	MAB1355	I
168Er	Ki67	DVS-Fluidigm	3168001B	I
169Tm	phospho-JAK2	CST	3776BF	I
170Er	DNMT3A	Novus	NB120-13888 (0.1 mg)	I
171Yb	CD68	DVS-Fluidigm	3171011B	I
172Yb	TET2	Proteintech	21207-1-AP (150 uL)	I
173Yb	PKCz	LSBio	LS-C354069 (100 uL)	I
174Yb	phospho-PKCz (T410)	Abclonal	AP0520 (100 uL)	I
175Lu	CD3	BioLegend	300443	S
209Bi	CD16	DVS-Fluidigm	3209002B	S
191Ir	Ir DNA-Intercalator	DVS-Fluidigm	201192A	nucleus
193Ir	Ir DNA-Intercalator	DVS-Fluidigm	201192A	nucleus
103Rh	Rh DNA-intercalator	DVS-Fluidigm	201103A	Viable cells

### hPBMC culture, irradiation or colchicine stimulation, CyTOF sample preparation and staining

The Institutional Review Board of The University of Texas MD Anderson Cancer Center approved the clinical study protocol (#PA16-0971). PBMCs were collected from a single healthy adult donor. PBMCs were isolated using Ficoll-Paque Plus (Fisher #45-001-750) according to the manufacturer's protocol. The Isolated PBMCs were resuspended in RPMI containing 5% pooled human serum (PHS), 10 mM HEPES, 0.2% gentamicin, and 2.5 U/mL benzonase nuclease to make a cell suspension containing 1.5 × 10^6^ cells/mL. The cells were divided equally into 12 of 35 mm plates (1.5 × 10^6^ cells/1.5 mL medium/plate) and were rested in an incubator for 1 h prior to treatment. These 12 plates corresponded to the four experimental conditions- non-IR -vehicle, non-IR -colchicine, IR-vehicle, IR-colchicine. Colchicine (10 μM) or vehicle stimulation was started 1 h before the irradiation, followed by IR (2 Gy), and the cells were incubated for 24 h. The cells were harvested and resuspended in MaxPar PBS (Fluidigm #201058). After washing with MaxPar PBS, the cells were applied to the viability staining with Cell-ID Intercalator-103Rh (Fluidigm #201103A) and washed with Maxpar PBS. For cell fixation, the cells were incubated with 1.6% formaldehyde. After washing two times with MaxPar PBS, the cells were resuspended in MaxPar Cell Staining Buffer (Fluidigm #201068) and were blocked by Human TruStain FcX (BioLegend #422302), followed by cell surface staining for cell type identification. After the wash with MaxPar PBS, the cells were chilled on ice and incubated at 4 ℃ with 1× saponin permeabilization buffer (eBiosciences #00-8333-56), followed by two washes with MaxPar PBS. The cells were resuspended in MaxPar Cell staining buffer and were incubated with metal-labeled antibodies against cytoplasmic proteins followed by three washes with MaxPar PBS. For the final fixation and nucleus staining, the cells were incubated with 1.6% formaldehyde containing Cell-ID Intercalator-Ir (Fluidigm #201192A) overnight at 4 ℃. 2103;. After washing twice with Maxpar Cell Staining Buffer and once with Maxpar water (Fluidigm #201069), the cells were filtered to make a single cell suspension and centrifuged. After adding EQ beads (Fluidigm #201078), CyTOF data were acquired on a Helios mass cytometer (Fluidigm).

### Irradiation

Cells were irradiated using an XRAD 320 x-Ray irradiator (Precision x-Ray/PerkinElmer), operating under standard conditions, which typically emits x-rays generated from a tungsten target at energies up to 320 kVp (kilovolt peak). A radiation dose of 2 Gy was delivered under these conditions. Dose rate at the irradiation height was measured using a calibrated ion chamber, following AAPM Task Group Report 61 guidelines. Calibration was performed by the MD Anderson Accredited Dosimetry Calibration Laboratory (ADCL), traceable to NIST standards (22).

### Data acquisition for CyTOF

CyTOF data were acquired on a Helios mass cytometer (Fluidigm) and were analyzed using the Astrolabe Cytometry Platform (Astrolabe Diagnostics, Inc.) as well as described in our previous paper (21). Single-cell data have been clustered using the FlowSOM R package (23) and labeled using the Ek'Balam algorithm (24). Cell subset definitions were followed as previously described (25, 26). Cluster labeling, method implementation, and visualization were done through the Astrolabe Cytometry Platform (Astrolabe Diagnostics, Inc.). All samples were acquired on the same day to minimize batch effects.

### Statistical analysis

The multidimensional scaling (MDS) map was generated using the cmdscale R package (27). Differential abundance analysis was done using the edgeR R package (28, 29) as previously described (30). Differential expression analysis was done using the limma R package (31), as described previously (32). Features with *P* values [or adjusted (Adj.) *p*-values] < 0.05 were considered statistically significant. For the Adj. *p*-value, limma R package is using the Benjamini-Hochberg correction. All samples originated from a single healthy donor. This experimental design was intentionally chosen to evaluate treatment-induced cellular responses within a consistent biological background, rather than to address inter-donor variability.

Statistical comparisons between two independent groups were performed using paired, two-tailed Student's t-tests. For analyses involving more than two groups, repeated measures one-way analysis of variance (ANOVA) followed by Tukey's multiple-comparisons test was applied. Normality of data distributions was assessed using the Shapiro–Wilk test. Statistical analyses were performed using GraphPad Prism (GraphPad Software, San Diego, CA, USA), and *P* values < 0.05 were considered statistically significant. The error bars in the scatter dot plots represent the mean ± SEM.

## Results

### The role of colchicine in modulating radiation-induced senescence and inflammation in immune cells

In this study, we isolated peripheral blood mononuclear cells (PBMCs) from a single healthy human donor and pre-treated these cells with colchicine before exposing them to 2 Gy of radiation as we described previously (22, 33). We then investigated the effects of colchicine pre-treatment on radiation-induced SASP. Using multiparameter mass cytometry (CyTOF), we evaluated the phenotypic changes of immune cells with general cell surface markers and various senescent markers, DNA damage response-related markers, efferocytosis, and clonal hematopoiesis driver- associated protein expression. Our aim was to elucidate the potential regulatory roles of colchicine in modulating the immune response to radiation.

**Figure 1:**
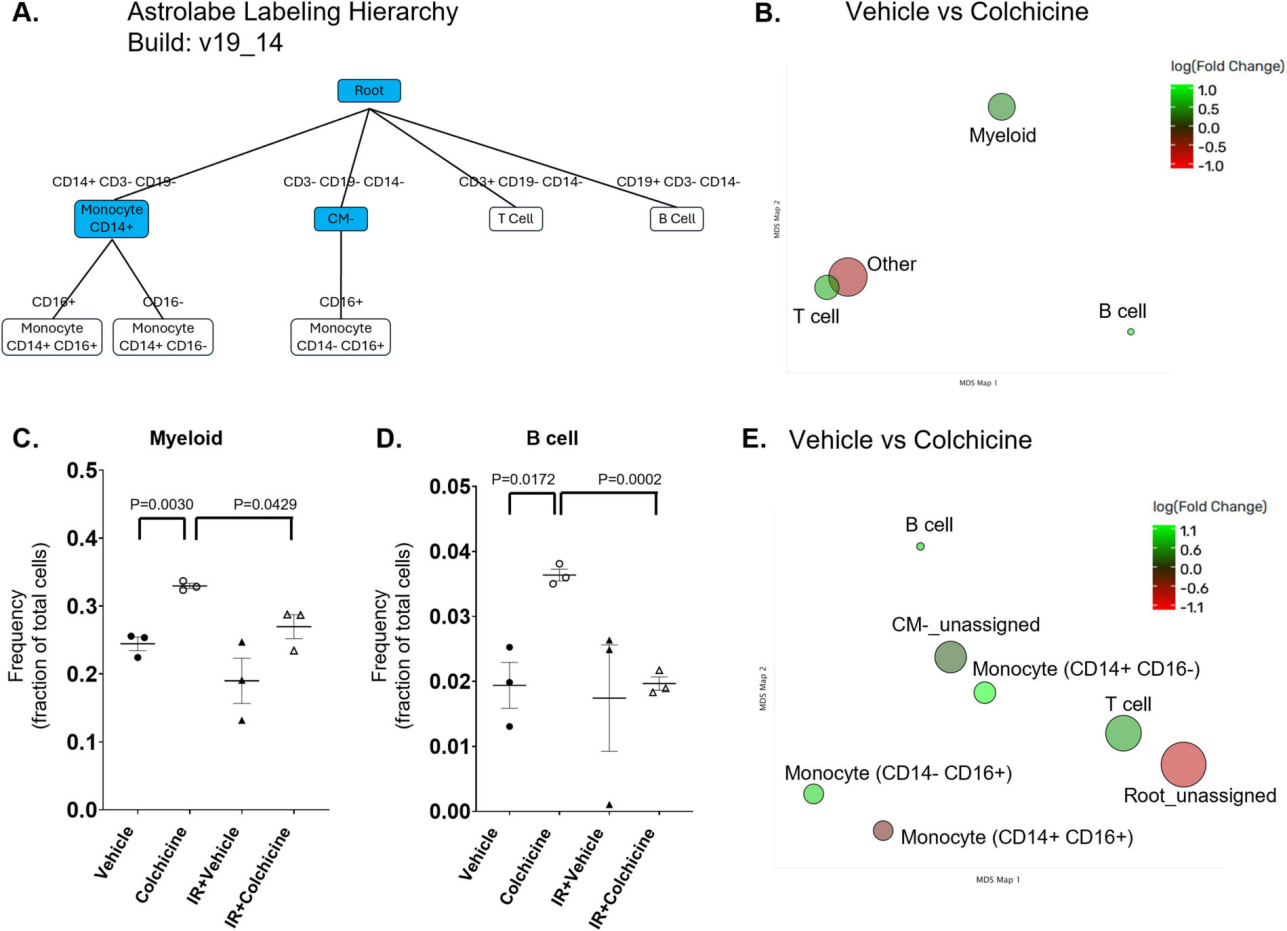
Cell subset using the astrolabe platform. The FlowSOM algorithm clustered cells and labeled subsets according to the canonical gating hierarchy. **(A)** Cell clustering and labeling with FlowSOM. Single-cell data were clustered using the FlowSOM algorithm, and clusters were organized into a hierarchical minimum spanning tree. Subsets were annotated according to the canonical immune cell gating hierarchy using the Astrolabe platform. **(B)** Differential abundance of immune cell populations visualized by MDS plot. Immune subsets were grouped into major compartments (T cells, B cells, myeloid cells, and other). Multidimensional scaling (MDS) was used to represent the similarity between populations in two-dimensional space. Bubble size corresponds to the relative frequency of each subset, while color indicates the log fold change (logFC) between conditions (green=increased, red=decreased). **(C, D)** Comparison of immune cell subset frequencies between groups. Frequencies of the immune cell subsets (T cells, B cells, myeloid cells, and other) were quantified, with statistical comparison showing a significant induction by colchicine in myeloid cells (*p* = 0.0030) **(C)** and B cells (*p* = 0.0172) **(D) (E)** Each bubble represents a major immune subset, positioned by MDS according to similarity in marker expression. Bubble size corresponds to subset frequency, while color indicates logFC between conditions (green=increased, red=decreased). B cells and monocyte subsets (CD14^+^CD16^−^, CD14^−^CD16^+^) were expanded, whereas the unassigned “Root” cluster was reduced.

**Figure 2:**
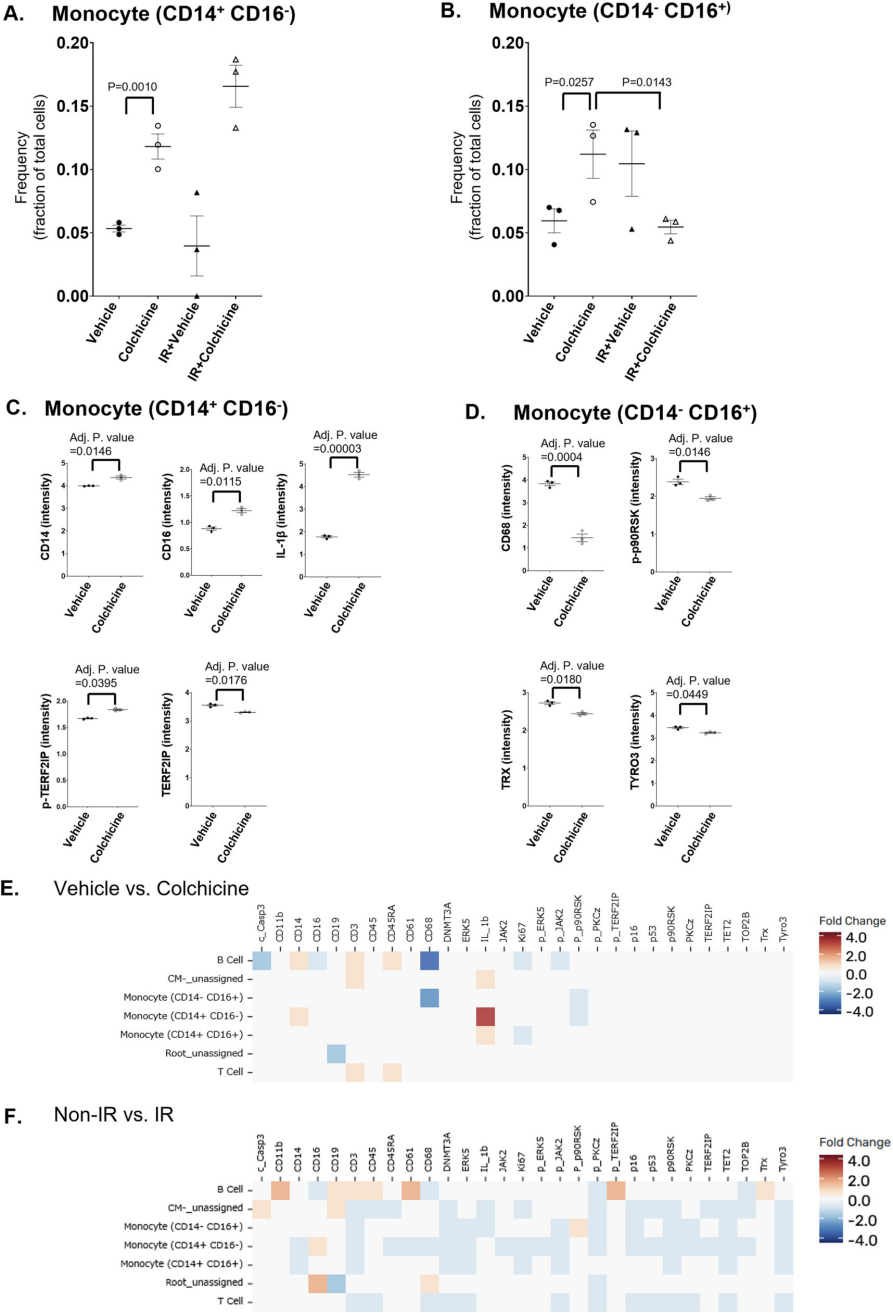
Differential abundance and marker expression of immune subsets across groups. **(A, B)** Frequencies of monocyte subsets across conditions. Scatter dot plots show the distribution of classical monocytes (CD14^+^ CD16^−^, left) and non-classical monocytes (CD14^−^ CD16^+^, right) as a fraction of total immune cells. Both subsets show condition-dependent changes, with expansion in specific groups compared to controls. **(C, D)** Expression intensity of immune markers in the monocyte subsets. Scatter dot plots show the expression levels (mean fluorescence intensity, MFI) of indicated molecules in **(C)** CD14^+^ CD16^−^ classical monocytes and **(D)** CD14^−^ CD16^+^ non-classical monocytes. Error bars indicate SEM across samples. **(E, F)** Heatmap of differential marker expression across immune subsets. Rows represent major immune cell populations, and columns represent measured molecules. **(E)** Vehicle vs. colchicine treatment. **(F)** Non-IR (vehicle) vs. IR-treated group. Color indicates fold change between the groups (red=upregulation, blue=downregulation).

Analysis using the Astrolabe platform (34) automatically labeled canonical immune cell subsets (Figures 1A, B). Total acquired events, Live single cells and 103Rh Live cell viability in our CyTOF experiment are also shown in Supplementary Table S1. Astrolabe identified three monocyte subsets, as well as B and T cell subsets. Although exposure to 2 Gy of IR did not increase any of these subsets, colchicine treatment significantly increased the myeloid and B cell subsets (Figures 1C–E). Specifically, colchicine increased the frequency of both monocyte subsets: CD14 ^+^ CD16^−^ and CD14^−^CD16^+^ (Figures 2A, B). CD14 ^+^ CD16^−^ monocytes, also known as classical monocytes, are the most abundant, making up about 80–90% of peripheral blood monocytes. They are primarily involved in phagocytosis. They also produce pro-inflammatory cytokines like TNF-α, IL-6, and IL-1β in response to infections. Classical monocytes can differentiate into macrophages and dendritic cells, playing a crucial role in initiating and regulating immune responses. CD14^−^CD16^+^ monocytes, or non-classical monocytes, patrol blood vessel walls and are involved in tissue repair and resolution of inflammation. They produce anti-inflammatory cytokines and are important for maintaining vascular homeostasis. These monocytes also respond to viral infections by producing type I interferons. In the CD14^+^CD16^−^ monocyte subset, colchicine treatment at the baseline condition led to a significant upregulation of CD14, CD16, IL-1β, and phosphorylated TERF2IP (p-TERF2IP), accompanied by a reduction in total TERF2IP expression (Figures 2C–E). These findings suggest that colchicine may promote inflammation and cellular senescence in this particular monocyte subset-an effect opposite to its reported anti-inflammatory actions in other cell types (20).

In contrast, following colchicine treatment, the CD14^−^CD16^+^ monocyte subset exhibited a significant reduction in the expression of CD68, phosphorylated p90 ribosomal S6 kinase (p-p90RSK), thioredoxin (TRX), and TYRO3 (Figures 2D, E). These markers are associated with monocyte activation, impaired anti-oxidant response, and immunoregulatory signaling. CD68 is a lysosomal glycoprotein commonly used as a marker of monocyte/macrophage activation. Its downregulation suggests a shift toward a less inflammatory phenotype. p-p90RSK, a downstream effector of the ERK/MAPK pathway, is involved in cell survival and inflammatory signaling; reduced phosphorylation indicates suppression of pro-inflammatory kinase activity. TRX, a key antioxidant protein, regulates redox balance and modulates inflammatory responses. Its decreased expression may reflect reduced oxidative stress or a dampened activation state. TYRO3, part of the TAM receptor family, contributes to immune homeostasis and efferocytosis. Lower TYRO3 levels may indicate altered monocyte differentiation or reduced engagement in tissue repair mechanisms. Together, these changes suggest that colchicine exerts a broad immunomodulatory effect on CD14^−^CD16^+^ monocytes, attenuating both inflammatory and oxidative signaling pathways. This supports its therapeutic potential in conditions characterized by monocyte-driven inflammation.

Importantly, while colchicine treatment led to an increase in IL-1β expression in both CD14^+^CD16^−^ and CD14^+^CD16^+^ monocyte subsets (Figures 2C–E), a distinct response was observed in the CD14^−^CD16^+^ monocytes. In this subset, colchicine significantly reduced the expression of CD68 and phosphorylated p90RSK (p-p90RSK) (Figures 2D, E). Furthermore, heatmap analysis revealed a clear distinction between non-irradiated (non-IR) and irradiated (IR) conditions, with a notable increase in p-p90RSK following IR exposure observed exclusively in the CD14^−^CD16^+^ monocytes (Figure 2E), unlike the other monocyte subsets. Additionally, only the CD14^−^CD16^+^ monocyte subset showed a trend toward increased frequency under IR conditions, which was reversed by colchicine treatment (Figures 2A, B). Based on these findings, we focused our investigation on the role of colchicine in modulating IR-induced responses specifically within the CD14^−^CD16^+^ monocyte subset.

### Radiation-induced changes in CD14-CD16^+^ monocytes: the role of p90RSK activation and SASP regulation

**Figure 3:**
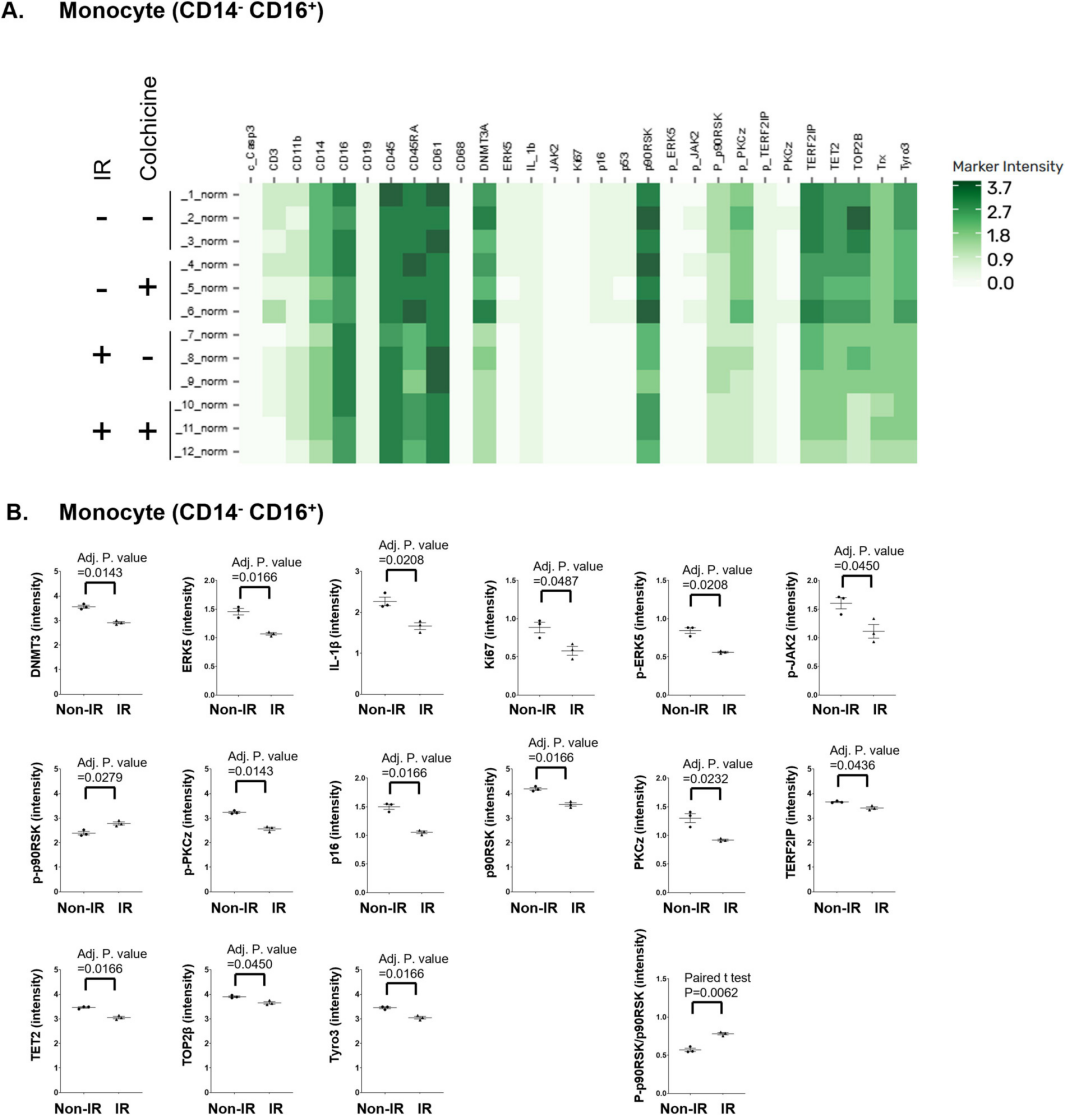
Heatmap of molecule expression across experimental groups in the monocyte subset (CD14^−^CD16^+^). **(A)** Expression levels of surface and signaling molecules in the monocyte subset (CD14^−^CD16^+^) were measured across four groups: Non-IR vehicle (samples 1–3), Non-IR colchicine (samples 4–6), IR vehicle (samples 7–9), and IR colchicine (samples 10–12). Darker green indicates higher expression intensity. Compared to the vehicle, colchicine treatment reduced the expression of several activation markers in both Non-IR and IR conditions. IR induced robust upregulation of phosphorylated p90RSK (p-p90RSK) activation in this monocyte subset. **(B)** Expression intensity of immune markers in the monocyte subset. Scatter dot plots show the expression levels (mean fluorescence intensity, MFI) of indicated molecules in CD14^−^ CD16^+^ non-classical monocytes. Error bars indicate SEM across samples.

Since only the CD14^−^CD16^+^ monocyte subset showed a trend toward increased frequency after IR in monocyte subsets, we focused on the expression changes in this subset. Our analysis revealed that IR specifically upregulated the expression of phosphorylated p90RSK (p-p90RSK). In contrast, the expression of other molecules, including those related to clonal hematopoiesis drivers (DNMT3A, TET2, p-JAK) and the senescence-associated secretory phenotype (SASP)-related molecules (p-ERK5, ERK5, p90RSK, p16, p-PKC*ζ*, PKC*ζ*, TERF2IP, TOP2b, and Tyro3), was significantly downregulated (Figure 3). We observed a significant increase in the ratio of p-p90RSK to p90RSK after IR, indicating enhanced activation of p90RSK. Additionally, protective molecules against SASP, such as TERF2IP, TOP2b, and Tyro3, were significantly decreased in this monocyte subset (Figure 3B). This suggests that p90RSK activation may play a role in promoting SASP after IR. Furthermore, the significant decrease in the expression of clonal hematopoiesis-associated molecules including DNMT3A and TET2 suggests a potential link between p90RSK-mediated SASP and clonal hematopoiesis-related pathways after IR in this monocyte subset. These findings indicate that IR-induced changes in CD14^−^CD16^+^ monocytes involve complex interactions between p90RSK activation, SASP, and clonal hematopoiesis-associated protein expression, highlighting the multifaceted impact of radiation on immune cell function.

### Functional profiling identifies a radiation-sensitive CD14^−^CD16^+^CD68^hi^ monocyte subset modulated by colchicine

**Figure 4:**
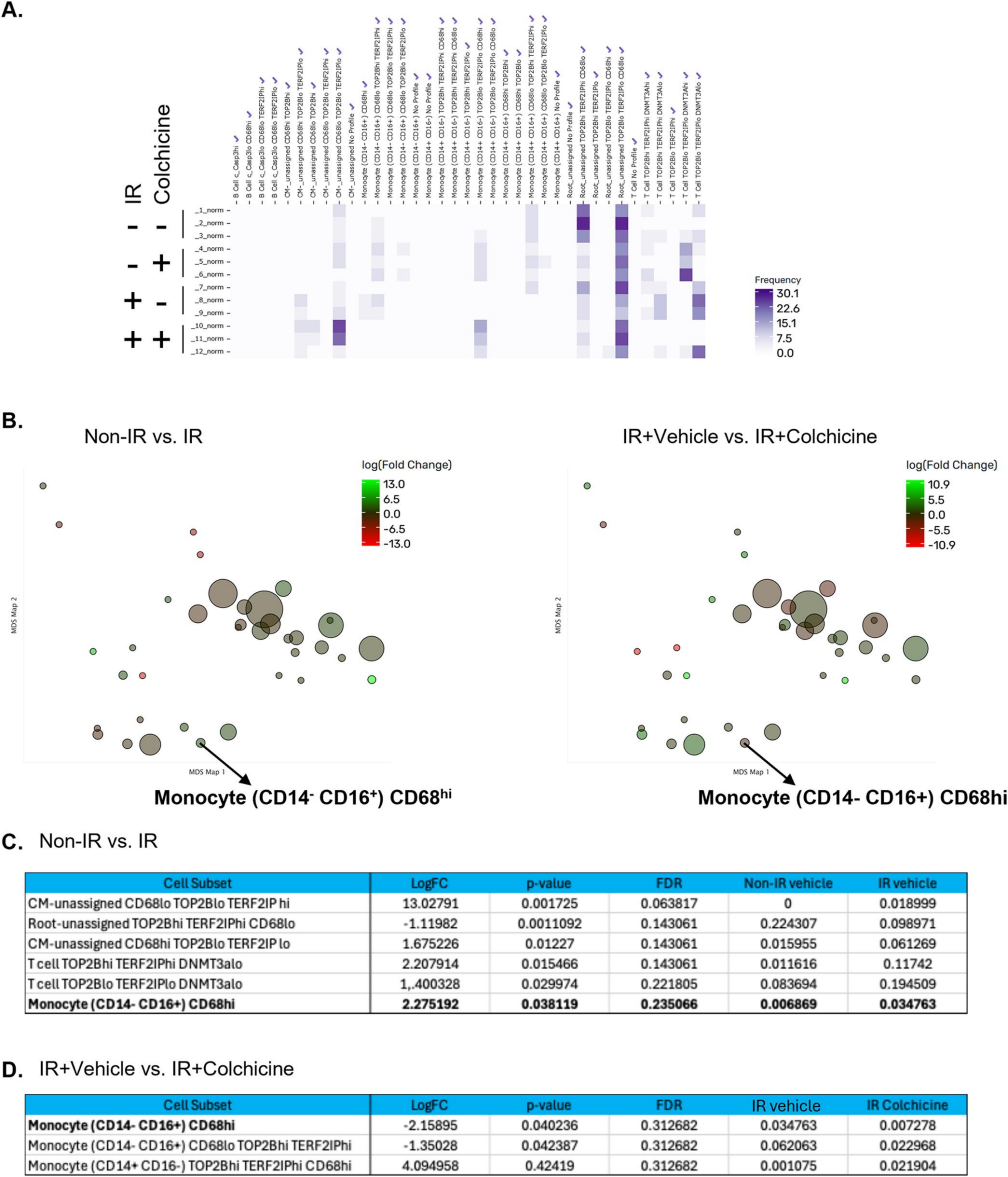
Colchicine alters gene expression in response to IR. **(A)** Heatmap of immune cell subset frequencies following ionizing radiation (IR) and colchicine treatment. Peripheral blood mononuclear cells (PBMCs) from a single healthy donor were pre-treated with colchicine and exposed to 2 Gy IR. After 24 h, cell populations were analyzed by CyTOF and annotated using the Astrolabe platform. The heatmap displays the relative frequency (%) of annotated immune subsets under four experimental conditions. Samples were divided into four groups: Non-IR vehicle (samples 1–3), Non-IR colchicine (samples 4–6), IR vehicle (samples 7–9), and IR colchicine (samples 10–12). Color intensity corresponds to cell frequency, with darker shades indicating higher abundance. Notably, IR increased the frequency of specific monocyte subsets, including CD14^−^CD16^+^CD68^hi^, whereas colchicine treatment reversed this expansion**. (B)** MDS plot of differential frequency analysis comparing (left) Non-IR vs. IR, and (right) IR + Vehicle vs. IR + Colchicine. Bubble size reflects frequency abundance, and color indicates direction of change (green=increased, red=decreased). **(C, D)** Differentially regulated cell subsets in response to IR and colchicine treatment. **(C)** Table summarizing cell subsets significantly altered (*p* < 0.05) between Non-IR vehicle and IR vehicle conditions. Shown are log₂ fold change, *p*-value, false discovery rate (FDR), and group-specific frequency values. **(D)** Table summarizing cell subsets significantly altered (*p* < 0.05) between IR + Vehicle and IR + Colchicine conditions. Colchicine-responsive changes in the context of IR are reported with log₂ fold change, *p*-value, FDR, and frequency.

**Figure 5:**
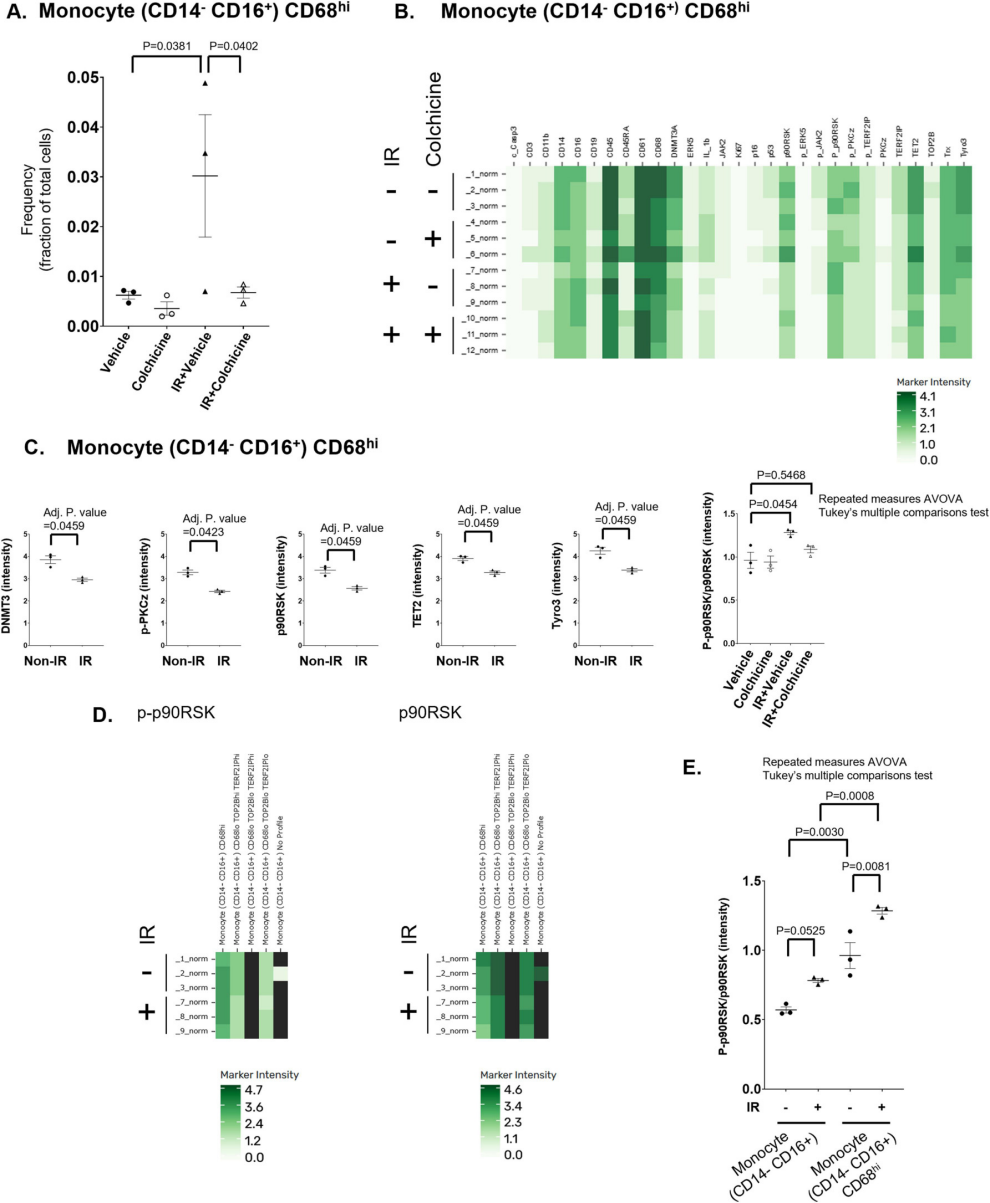
Frequency of CD14^−^ CD16^+^ CD68^hi^ monocytes across treatment groups. **(A)** Scatter dot plot showing the frequency of CD14^−^ CD16^+^ CD68^hi^ monocytes in Non-IR vehicle, Non-IR colchicine, IR vehicle, and IR colchicine groups. **(B)** Expression levels of surface and signaling molecules in the monocyte subset (CD14^−^ CD16^+^) CD68^hi^ were measured across four groups: Non-IR vehicle (samples 1–3), Non-IR colchicine (samples 4–6), IR vehicle (samples 7–9), and IR colchicine (samples 10–12). Darker green indicates higher expression intensity. **(C)** Expression intensity of the markers which had significant change by IR in the monocyte subset (CD14^−^ CD16^+^) CD68^hi^. The ratio of phosphorylated p90RSK to total p90RSK (p90RSK activity) was induced in IR vehicle group, but not in IR colchicine group compared to Non-IR vehicle group in the monocyte subset (CD14^−^ CD16^+^) CD68^hi^. Scatter dot plots show the expression levels (mean fluorescence intensity, MFI) of indicated molecules. Error bars indicate SEM across samples. **(D)** Expression of phosphorylated and total p90RSK in the CD14^−^ CD16^+^ monocyte subsets. Heatmaps depicting marker-defined heterogeneity of CD14^−^ CD16^+^ monocytes. (Left) Relative expression of phosphorylated p90RSK (p-p90RSK), reflecting kinase activation status. (Right) Relative expression of total p90RSK protein. Color intensity (green scale) indicates marker expression levels. These data demonstrate that phosphorylation-specific changes in p90RSK do not always mirror total protein levels, highlighting dynamic regulation of this signaling pathway in CD14^−^ CD16^+^ monocyte subsets under IR treatment. **(E)** p90RSK activity in CD14^−^ CD16^+^ monocytes and the CD68^hi^ subset. Scatter dot plot showing relative p90RSK activity across treatment groups in (left) the broad CD14^−^ CD16^+^ monocyte subset and (right) the CD14^−^ CD16^+^ CD68^hi^ monocyte subset. IR increased p90RSK activity in both subsets (IR-induced p90RSK activity in CD14^−^ CD16^+^ monocytes was also displayed in Figure3B). The CD14^−^ CD16^+^ CD68^hi^ subset exhibited higher activation compared to the broader subset, suggesting this subset is particularly sensitive to IR-induced signaling and colchicine modulation.

We next incorporated functional antibody staining data, and analysis through the Astrolabe platform identified 37 distinct functional profiling subsets, which were annotated based on the markers most critical for their differentiation (Figures 4A, B). Among these, a specific monocyte subset (CD14^−^CD16^+^CD68^hi^) showed a significant increase in frequency following IR exposure, an effect that was reversed by colchicine treatment. Notably, this was the only subset in which colchicine shifted the IR-induced regulatory response in the opposite direction (Figures 4C, D, 5A).

Within the CD14^−^CD16^+^CD68^hi^ monocyte subset, IR resulted in downregulation of CHIP-associated protein expression, including DNMT3A and TET2, accompanied by increased activation of p90RSK (the ratio of p-p90RSK to p90RSK) (Figures 5B, C). This subset represents non-NK, non-classical patrolling monocytes with anti-inflammatory characteristics. The combined effects of IR and colchicine suggest that colchicine acts beyond its canonical anti-inflammatory role, particularly by modulating radiation-induced macrophage activation. Importantly, this subset consistently exhibited higher p90RSK activity compared to the broader CD14^−^CD16^+^ monocyte subset under both basal and IR conditions (Figures 5D, E). These findings support the concept that p90RSK activity in CD14^−^CD16^+^CD68^hi^ monocytes is more sensitive to IR-driven stress responses and to colchicine-mediated effects than other monocyte subsets, potentially contributing to the persistence of monocyte senescence.

Notably, this subset showed a significant increase in frequency following IR-a change not observed in other monocyte subsets (Figures 4C, D, 5A). Colchicine treatment reversed this increase, restoring the subset's frequency to baseline levels prior to IR. This unique response underscores both the distinct sensitivity of this monocyte subset to IR and the specific role of colchicine in modulating its behavior after irradiation.

## Discussion

In this study, we examined the effects of colchicine on IR-induced changes in senescence markers, DNA damage response, efferocytosis, and CH driver associated proteins in immune cells. Although IR did not alter frequency of total immune cell per total cell, colchicine increased the relative abundance of myeloid and B cell subsets. Within CD14^−^CD16^+^ monocytes, IR enhanced p90RSK activation despite concurrent reductions in proliferation, inflammatory, and oncogenic signaling markers such as p-JAK2 and p-PKC*ζ*. The selective expansion of the CD14^−^CD16^+^ CD68^hi^ monocyte subset and its heightened p90RSK activity suggest that colchicine may exert its immunomodulatory effects by targeting this uniquely responsive population. By inhibiting p90RSK-driven signaling in this unique monocyte subset, colchicine may counteract radiation-induced immune remodeling associated with reduced CH driver associated proteins expression including DNMT3A and TET2 and heightened stress-responsive signaling. *In vitro* this mechanism offers a potential pathway through which IR-induced immune dysfunction and stress responsive signaling.

It is important to note that these findings reflect early immune remodeling. In this study, we exposed PBMCs to a single dose of ionizing radiation (2 Gy) and assessed them 24 h later. The 2 Gy dose was intentionally chosen because it represents a clinically relevant, sublethal radiation exposure commonly used as a single fraction in conventional radiotherapy. Importantly, at this dose, the majority of immune cells do not undergo acute cell death and survive irradiation. This survival is critical to our study design, as it allows investigation of radiation-induced functional and transcriptional changes in viable immune cells rather than acute cytotoxic effects. The 24-hour time point was selected to capture early yet sustained cellular responses after radiation exposure, at a stage when surviving cells have recovered from immediate DNA damage signaling yet remain transcriptionally reprogrammed. Most early effects of radiation, such as activation of the DNA damage response, occur quickly and fade within a few hours; therefore, finding inflammatory or senescence-related markers at 24 h indicates that the cells have not fully recovered from the initial stress. While clinical radiotherapy often involves fractionated or repeated exposures, our study was designed to isolate the direct effects of a single clinically relevant dose, allowing clearer mechanistic insight into how radiation-exposed surviving immune cells may contribute to sustained immune dysfunction and stress-responsive signaling. The goal was not to mimic chronic radiation exposure, but to determine whether a single dose could trigger early and lasting immune responses in PBMCs.

The CH driver genes examined in this study were selected based on substantial prior evidence supporting their roles in atherosclerosis and cardiovascular disease. Numerous studies have investigated the functional consequences of CH driver gene loss using knockout mouse models and other animal systems. Collectively, these studies demonstrate that loss-of-function of key CH driver genes leads to a pro-atherosclerotic phenotype, establishing a mechanistic link between clonal hematopoiesis–related gene dysfunction and atherosclerosis.

Our study is therefore grounded in these well-established findings from CH driver gene knockout models, rather than focusing exclusively on mutation-driven mechanisms. Building on this framework, we demonstrate that radiation therapy can induce not only somatic alterations associated with clonal hematopoiesis, but also significant changes in the expression levels of CH driver genes. Importantly, these expression-level changes represent an additional mechanism by which CH drivers may contribute to cardiovascular disease risk, independent of overt loss-of-function mutations. Given that radiation-associated cardiovascular events have been extensively reported in prior studies, our findings suggest that radiation-induced dysregulation of CH driver gene expression may play a meaningful role in the development of cardiovascular disease.

The CD14^−^CD16^+^CD68^hi^ monocyte subset represents a specialized population of non-classical, patrolling monocytes with distinct functional and molecular characteristics. These cells are typically involved in vascular surveillance and the clearance of apoptotic cells, contributing to tissue homeostasis and anti-inflammatory responses (35, 36). The high expression of CD68, a marker associated with lysosomal activity and phagocytosis, suggests that this subset is primed for efferocytosis and may play a role in remodeling damaged tissue. In the context of IR, this subset becomes particularly relevant (37–39). IR exposure significantly expands the CD14^−^CD16^+^CD68^hi^ subset and induces p90RSK activation, a kinase implicated in regulating senescence-associated stemness and the senescence-associated secretory phenotype (SASP). These processes are central to chronic inflammation and tissue dysfunction following radiation injury. Importantly, this monocyte subset shows reduced expression of CHIP driver-associated proteins, including DNMT3A and TET2, potentially linking it to clonal hematopoiesis (CH)-a condition associated with increased cardiovascular disease (CVD) risk due to the expansion of hematopoietic clones with pro-inflammatory potential.

Colchicine, a microtubule-disrupting anti-inflammatory agent, selectively suppresses the IR-induced expansion of CD14^−^CD16^+^CD68^hi^ monocytes without restoring DNMT3A or TET2 levels. This suggests that colchicine may exert its protective effects by modulating p90RSK-driven signaling in this uniquely responsive subset. By inhibiting the expansion and activation of these monocytes, colchicine could potentially reduce IR-induced vascular inflammation and senescence, thereby mitigating the progression of CVD in individuals exposed to radiation or those with underlying CH. This mechanistic insight positions the CD14^−^CD16^+^CD68^hi^ monocyte subset as a potential biomarker and therapeutic target in radiation-associated cardiovascular pathology, especially in settings where immune senescence and clonal hematopoiesis intersect to drive disease progression.

In conclusion, while colchicine is well known for its broad anti-inflammatory effects-particularly its inhibition of NLRP3 inflammasome assembly and modulation of neutrophil activity (40)—its role in regulating the SASP and clonal hematopoiesis (CH) drivers has not been thoroughly investigated. In this study, we identified a novel function of colchicine in suppressing the induction of a distinct monocyte subset, CD14^−^CD16^+^CD68^hi^, which is characterized by elevated p90RSK activation following IR. Given our previous findings that p90RSK activation is closely linked to mitochondrial ROS production and persistent SASP signaling, this subset may play a key role in sustaining premature senescence and inflammatory responses after cancer therapies. These results underscore the importance of chronic SASP induction in contributing to CVD among cancer survivors. Recent studies provide additional evidence for an association between colchicine treatment and the modulation of inflammatory programs linked to clonal hematopoiesis, highlighting the potential significance of colchicine in mitigating CH-related cardiovascular risk (41).

Importantly, although the colchicine concentrations used in this study are higher than those achieved with low-dose colchicine therapy, even when considering the tendency of colchicine to accumulate in leukocytes, our findings suggest that colchicine's therapeutic potential may extend beyond conventional anti-inflammatory mechanisms. Since broad anti-inflammatory strategies can impair immune surveillance and potentially promote cancer recurrence or metastasis, alternative approaches are needed. We propose that targeting the mitochondrial-nuclear positive feedback loop, which drives p90RSK-mediated SASP signaling, could offer a promising strategy to reduce CVD risk in cancer survivors-even long after the completion of therapy.

This study has several limitations. First, the experiments were performed using samples from a single healthy donor and were conducted *in vitro*. Second, in our study, we observed an increase in cellular debris during *in vitro* experiments. This phenomenon may be attributed to the culture conditions of PBMCs. PBMCs are inherently sensitive to environmental stressors, and prolonged culture or suboptimal conditions can increase cell death and fragmentation, thereby contributing to debris accumulation. While this does not appear to affect the overall interpretation of our findings, it highlights the importance of optimizing culture protocols to minimize confounding effects in future mechanistic studies. Third, we used a single clinically relevant radiation dose and assessed outcomes at a single time point (24 h). Although we observed persistent senescence, inflammation, and changes in CHIP-associated protein expression, including DNMT3A and TET2, these findings do not address long-term radiation-induced cardiovascular effects in cancer survivors. Moreover, these experimental models do not fully represent the complexity of the *in vivo* environment or allow evaluation of changes in clonal expansion. Although we observed a decrease in CHIP-associated protein levels, we cannot directly correlate these changes with CHIP mutations or clonal expansion. Taken together, these limitations point to the need for future studies that comprise detailed molecular mechanistic analyses and *in vivo* models.

## Data Availability

The original contributions presented in the study are included in the article/Supplementary Material, further inquiries can be directed to the corresponding author.
