# Supplementary material for: Ionizing radiation expands a p90RSK-activated patrolling monocyte subset: modulation by colchicine
Source: Front Cardiovasc Med. 2026 Apr 15;13:1763490. doi: 10.3389/fcvm.2026.1763490 (PMC13124628; doi:10.3389/fcvm.2026.1763490)
Supplement: Supplementary file 1 [file Table1.docx]

**Supplementary Table S1.** Total acquired events, Live single cells and 103Rh Live cell viability in our CyTOF experiment.

| Average±SD | **None** | **Colchicine** | **IR** | **IR+Colchicine** |
| --- | --- | --- | --- | --- |
| Total acquired events | 111068 ± 38563 | 338311 ± 85054 | 142055 ± 53898 | 262496 ± 45495 |
| Live single cells | 54471 ± 19981 | 240114 ± 68633 | 51294 ± 26531 | 130266 ± 33853 |
| 103Rh Live cell viability (%) | 89.3 ± 0.3 | 97.4 ± 0.3 | 78.4 ± 3.4 | 88.7 ± 1.3 |
